# Changes in health complaints after removal of amalgam fillings

**DOI:** 10.1111/j.1365-2842.2011.02223.x

**Published:** 2011-11

**Authors:** T T Sjursen, G B Lygre, K Dalen, V Helland, T Lægreid, J Svahn, B F Lundekvam, L Björkman

**Affiliations:** * Department of Clinical Dentistry, University of BergenBergen, Norway; † Dental Biomaterials Adverse Reaction Unit, Uni HealthBergen, Norway; ‡ Department of Biological and Medical Psychology, University of BergenBergen, Norway

**Keywords:** health complaints, amalgam, before-and-after study, dental, restoration

## Abstract

The aim of the present study was to investigate whether removal of all amalgam fillings was associated with long-term changes in health complaints in a group of patients who attributed subjective health complaints to amalgam fillings. Patients previously examined at the Norwegian Dental Biomaterials Adverse Reaction Unit were included in the study and assigned to a treatment group (*n* = 20) and a reference group (*n* = 20). Participants in the treatment group had all amalgam fillings replaced with other restorative materials. Follow-ups took place 3 months, 1 and 3 years after removal of all amalgam fillings. There was no intervention in the reference group. Subjective health complaints were measured by numeric rating scales in both groups. Analysis of covariance was used to compare changes in health complaints over time in the two groups. In the treatment group, there were significant reductions in intra-oral and general health complaints from inclusion into study to the 3-year follow-up. In the reference group, changes in the same period were not significant. Comparisons between the groups showed that reductions in intra-oral and general health complaints in the treatment group were significantly different from the changes in the reference group. The mechanisms behind this remain to be identified. Reduced exposure to dental amalgam, patient-centred treatment and follow-ups, and elimination of worry are factors that may have influenced the results.

## Introduction

For decades, dental amalgam has been extensively used in the treatment of caries lesions. Dental amalgam consists of approximately 50% metallic mercury mixed with an alloy mainly consisting of silver, tin and copper (1). The safety of dental amalgam has been questioned, and it has been discussed to what extent mercury released from amalgam fillings may lead to adverse health effects (2–8). Generally, no deleterious effects from amalgam are detected in studies on samples of the general population (5, 9–11), and no adverse reactions could be detected in two randomised controlled studies on school children treated with dental amalgam (3, 4). Dental amalgam fillings release elemental mercury vapour in the mouth, resulting in elevated concentrations of mercury in blood, plasma and urine, and concentration of inorganic mercury in the brain (12–19). The possibility that a small fraction of the population may have predispositions to rare adverse reactions to dental amalgam cannot be ruled out; thus, research on adverse effects associated with exposure to dental amalgam should focus on the possibility of rare outcomes (20). People with health complaints attributed to dental amalgam believe their health complaints are caused, or aggravated, by mercury released from their amalgam fillings. It has been established that dental amalgam fillings may lead to local adverse reactions, including oral lichenoid reactions (21), and removal of amalgam fillings in contact with the lesions is generally recommended. However, for a number of patients, no objective signs of adverse reactions to amalgam fillings, or other diseases explaining their complaints, can be observed (22). Patients who attribute subjective health complaints to dental amalgam describe a number of health complaints including tiredness, headaches, pain from muscles and joints, and problems with memory and concentration (18, 22). There is a lack of treatment options for patients without objective signs of adverse reactions to amalgam fillings, and removal of sound amalgam fillings is generally not recommended. Some patients nevertheless decide to remove all amalgam fillings at their own initiative (23), and studies have reported significant improvements in subjective health complaints after the removal of amalgam fillings (24, 25).

The aim of the present study was to investigate whether removal of all amalgam fillings in a group of patients who attributed subjective health complaints to dental amalgam (treatment group) was associated with long-lasting changes in subjective health complaints. The underlying null hypothesis was that there would be no significant differences in long-term changes in health complaints between the treatment group and a comparable reference group. In addition, secondary analyses of changes in health complaints in the treatment group and the reference group were investigated independently, testing the null hypotheses of no changes in health complaints within each group. Within-group changes in mercury concentration in serum and urine in the treatment group were also investigated.

## Materials and methods

### Design

The study was designed as a before-and-after study with a comparison group (reference group) comparing changes in health complaints in a treatment group, which had all amalgam fillings replaced with other restorative materials, with changes in health complaints in a comparable reference group, which did not receive any intervention.

### Participants

**Fig. 1:**
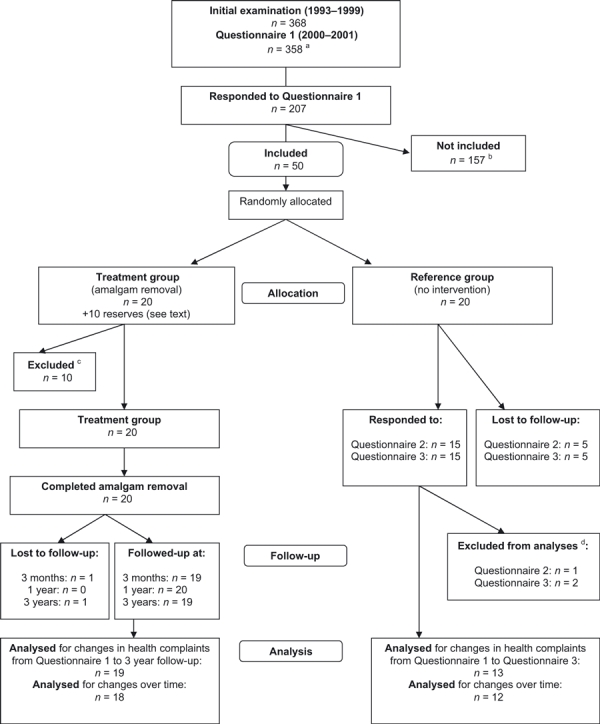
Participant flow. Flow diagram showing participant flow in the study. The study is a before-and-after study with a comparison group (reference group). ^a^Current addresses were missing for 10 patients; ^b^did not fulfil inclusion criteria listed in Table 1; ^c^excluded according to exclusion criteria listed in Table 1; ^d^removed all amalgam fillings.

Participants were recruited from patients (*n* = 368) examined at the Norwegian Dental Biomaterials Adverse Reaction Unit in the period 1993–1999 (initial examination; Fig. 1). The majority of the patients had been referred to the unit because of health complaints attributed to amalgam fillings (22). Generally, either the patient or the referring physician/dentist had raised the question that dental materials could be a causal or contributing factor related to the patient's health problems. In 2000–2001, patients with known addresses (*n* = 358) were sent a questionnaire (Questionnaire 1) regarding current health complaints and medical and dental treatment since the initial examination. The questionnaire was returned by 207 patients (Fig. 1). Based on the responses to the questionnaire, 157 patients did not fulfil one or more of the inclusion criteria listed in Table 1, leaving 50 patients who were randomly allocated into a treatment group (*n* = 20), a reference group (*n* = 20) and a group of reserves (*n* = 10; Fig. 1). The function random number in Microsoft Excel 97 was used for the allocation. The exclusion criteria listed in Table 1 were applied to the treatment group in order to increase the probability of participants in this group being able to complete the replacement process. Six participants were excluded from the treatment group according to these criteria. The same exclusion criteria were used in the group of reserves for sequential inclusion into the treatment group, resulting in four participants not being eligible for participation in the treatment group. The remaining six participants from the group of reserves were used to replace the excluded participants from the treatment group. The criteria were applied based on clinical documentation, telephone interviews and a clinical examination (pre-treatment examination). The exclusion criteria were initially not applied to the reference group as no intervention was planned for this group.

**Table 1:** Eligibility criteria and number of patients not included. Inclusion criteria were applied based on information from initial examination and Questionnaire 1. Exclusion criteria were applied in the treatment group and reserves, and were applied in relation to the pre-treatment examination in September 2000

Inclusion criteria	Numbers not fulfilling criterion†
Referred to the Norwegian Dental Biomaterials Adverse Reaction Unit for examination of health complaints attributed to amalgam fillings	33
Amalgam fillings still present	79
No diagnosed contact allergy to substances in resin-based dental materials	54
Health complaints from at least three different organ systems	25
Data on mercury in blood and urine from initial examination	59
Age 25–55 at initial examination	10
Accepted to be contacted in a follow-up study	11

Exclusion criteria (treatment group and reserves)	Numbers excluded
Severe medical disorders (e.g. multiple sclerosis, ALS, severe rheumatoid arthritis)	1
Severe food allergies	1
Psychological difficulties or psychiatric disorders that could influence the dental treatment	3
Complicated therapy (severe periodontitis, high caries activity and/or need for complicated dental rehabilitation – e.g. bridges)	4
Inclusion criteria no longer fulfilled	1‡

† One hundred and fifty-seven patients did not fulfil one or more of the inclusion criteria.

‡ Completed removal of amalgam fillings since responding to Questionnaire 1.

### Initial examination (1993–1999)

At the initial examination at the unit (22), patients underwent a medical and dental examination. Blood and urine samples were collected and analysed for mercury in addition to routine analyses (17). Patients were also asked to complete questionnaires regarding suspected adverse reactions to dental materials, current and previous health complaints and demographic variables. Participants included in the present study had at the initial examination neither signs of contact allergic reactions to dental materials nor a known history of such reactions and consequently were not recommended removal of amalgam fillings.

### Questionnaire 1 (2000–2001)

Questionnaire 1 included questions regarding current health complaints, treatment since the initial examination and demographic variables. Health complaints were measured by numeric rating scales using numbers from 0 to 10. No information on a planned intervention study was given in the questionnaire. Responses to Questionnaire 1 were used for identifying patients eligible for participation and as baseline values for comparisons of changes in health complaints in the treatment group and the reference group. Questions from Questionnaire 1 were included in all subsequent questionnaires.

### Pre-treatment examination

In September 2002, participants in the treatment group underwent a pre-treatment examination consisting of medical and dental examinations and collection of samples of blood serum and urine. Blood serum was analysed for mercury concentration by sector field inductively coupled plasma–mass spectrometry (26, 27), while urine was analysed for mercury concentration by cold vapour atomic absorption spectrometry (28). Participants also responded to a questionnaire similar to Questionnaire 1. The pre-treatment examination and all subsequent follow-ups took place at the Dental Biomaterials Adverse Reaction Unit. Participants in the reference group were not assigned any treatment and were not asked to go through a pre-treatment examination.

### Intervention

The assigned intervention in the treatment group was removal of all amalgam fillings. The amalgam fillings were replaced with other dental restorative materials (e.g*.* composites, ceramic restorations and metalloceramic crowns). All treatment costs were covered by project funds. Replacement of amalgam fillings is not possible to mask, and thus, no blinding was used. The replacement was carried out by the participants’ own dentists according to clinical guidelines aiming at minimal exposure to mercury during removal sessions (29). The dentists were instructed to use rubber dam, high-volume suction, water cooling and to remove fillings in chunks using a sharp dental bur. Eighteen dentists from 18 different dental practices were involved in the study. One dentist treated three patients; the other dentists treated one patient each. Participants were given written instructions to contact the Dental Biomaterials Adverse Reaction Unit if they experienced increased health complaints like chills, fever, pain and rashes in relation to the amalgam replacement process. These instructions included advice to the patient's physician regarding blood tests to be taken (leucocytes, CRP, IgE and mercury concentration in blood) in case of increased health complaints after dental treatment.

To compare replacement of amalgam fillings with the standard treatment (i.e*.* no amalgam replacement), no intervention was assigned to the reference group.

### Follow-up

#### Treatment group

**Fig. 2:**
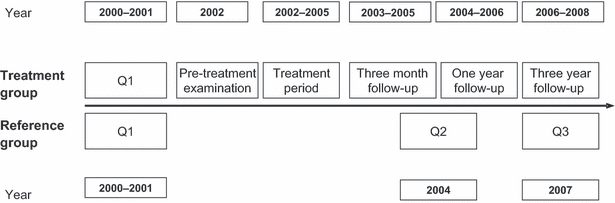
Timeline for the study. Timeline for the trial for the treatment group and the reference group. Q1, Q2 and Q3 indicate Questionnaire 1, Questionnaire 2 and Questionnaire 3, respectively. Time frames for the activities are indicated for the treatment group (top) and the reference group (bottom).

Routines for the follow-ups were similar to the pre-treatment examination. Follow-ups took place 3 months, 1 and 3 years after completed replacement of amalgam fillings (Fig. 2). The follow-ups included control of the new dental restorations by a dentist, questions about experienced side effects like post-operative dental pain and other complications, and collection of serum samples. Urine samples were collected at follow-up after 1 year. No general medical interview or health guidance was included.

#### Reference group

Follow-ups in this group were limited to questionnaires sent by post. Participants were sent Questionnaire 2 in 2004 and Questionnaire 3 in 2007. Questionnaire 2 was given at approximately the same time as the majority of participants in the treatment group went through their 1-year follow-up. Questionnaire 3 was given in parallel with the 3-year follow-up in the treatment group (Fig. 2). Based on available information from the initial examination and Questionnaire 1, the exclusion criteria used in the treatment group were applied *post hoc* to the reference group, resulting in two of the initial 20 participants being excluded. Reasons for exclusion were severe food allergy and complicated dental treatment (one patient) and diagnosed contact allergy to substances in resin-based dental materials (one patient). Results from comparisons of changes in health complaints in the treatment group and the reference group were calculated using both the initial reference group and the reference group with the two participants excluded from analyses.

### Outcome variables

Primary outcome measures were changes in local oro-facial complaints and general health complaints from Questionnaire 1 (inclusion into study) to the 3-year follow-up in the treatment group and to Questionnaire 3 in the reference group. Current health complaints in both groups were measured by numeric rating scales (30) included in the questionnaires. The questionnaires were given at all measure points. The same scales have previously been used in a similar patient population (8) and include 23 items addressing a diverse range of oro-facial and general health complaints frequently reported by patients with subjective health complaints attributed to amalgam fillings. Oro-facial complaints were categorised as either intra-oral (six items: intra-oral burning sensation, intra-oral pain/tenderness, taste disturbances, intra-oral stiffness/paresthesia, dry mouth and increased salivation/mucus) or extra-oral (five items: extra-oral burning sensation, extra-oral pain/tenderness, extra-oral stiffness/paresthesia, extra-oral skin problems and pain from temporomandibular joints). The sum scores for each category were used as index scores (8). Index scores for general health complaints (12 items: musculoskeletal complaints, gastrointestinal complaints, cardiovascular complaints, skin problems, complaints related to eyes/sight, complaints related to ears/hearing/nose/throat, tiredness, dizziness, headaches, memory problems, difficulty concentrating and anxiety/depression) were constructed in the same way (8). Highest possible index score was 60 for intra-oral index, 50 for extra-oral index and 120 for the general health complaints index. Internal consistency for the indices was estimated by Cronbach's alpha using the entire group of patients randomised (*n* = 50; Fig. 1) and found to be 0·66, 0·72 and 0·80, respectively.

### Power calculation

Number of participants included in this study was limited by available patients. One of the main objectives of the study was to test the null hypothesis that changes in index scores for health complaints were equal in the treatment group and in the reference group. Assuming a mean difference in index score for general health complaints of 10·0 between the groups (corresponding to a mean difference before–after of 10·0 in the treatment group versus a mean difference of 0·0 in the reference group) and a common within-group standard deviation of 10·0, a sample size of 20 patients in each group will give the study a power of 87% to yield a statistically significant result. The criterion for significance (alpha) was 0·05, and the test was two-tailed.

### Statistical methods

Mean values with 95% confidence intervals and analysis of variance were used for comparisons between groups. Paired-sample *t*-tests and analysis of variance for repeated measures were used to investigate within-group changes over time. Variables for changes in health complaints, from Questionnaire 1 to the 3-year follow-up in the treatment group and from Questionnaire 1 to Questionnaire 3 in the reference group, were constructed by subtracting the most recent scores from the scores from Questionnaire 1. A positive value indicated a reduction in complaints, whereas a negative value indicated increased complaints. The primary hypothesis of changes in reported health complaints in the treatment group compared with the reference group was tested by between-group comparisons of unadjusted pre–post per-protocol changes in the two groups using independent-sample *t*-tests. Adjustments for age, gender, and complaint intensity reported in Questionnaire 1 were made by analysis of covariance. We used last value carried forward to replace missing values for intention-to-treat analysis (ITT). SamplePower 2.0* was used for power calculations, and SPSS 15.0* was used for all other statistical analyses. *P*-values <0·05 were considered statistically significant for all analyses.

### Ethical approval and registration

The project protocol was approved by the Regional Committee for Medical Research Ethics in Western Norway (REK III, 24.01) and registered at ClinicalTrials.gov (NCT00346944). Participants in the treatment group received information on possible side effects from new fillings and possible post-operative complications following replacement of amalgam fillings. Written consent was obtained from all participants in both groups.

## Results

### Participant flow and numbers analysed

#### Treatment group

All 20 participants in the treatment group received the assigned intervention (replacement of all amalgam fillings). One participant could not attend the 3-month follow-up, and another participant could not attend the 3-year follow-up. For analysis of changes in health complaints from Questionnaire 1 to the 3-year follow-up, data from 19 participants were analysed (Fig. 1). For repeated measures analysis, data from 18 participants were analysed (Fig. 1).

#### Reference group

Questionnaire 2, which was sent to all 20 participants in the reference group in 2004, was returned by 15 participants. One participant reported having removed all amalgam fillings between Questionnaire 1 and Questionnaire 2. Questionnaire 3 was sent to all 20 participants in the reference group. The questionnaire was returned by 15 participants (Fig. 1). For analyses of changes in health complaints from Questionnaire 1 to Questionnaire 3, data from 13 participants were analysed. For repeated measures analysis, data from 12 participants were analysed. Changes in health complaints in the treatment group were also compared with changes in the reference group after *post hoc* application of exclusion criteria based on data from 12 participants in the reference group.

### Initial examination and Questionnaire 1

Data from the initial examination and Questionnaire 1 were used as baseline values in the study. Number of amalgam surfaces and concentration of mercury in blood and urine were not significantly different between the groups at the initial examination (Table 2). Results from Questionnaire 1 showed that the final treatment group (*n* = 20) was similar to the reference group (*n* = 20) with regard to age, gender distribution, education level and medication. Levels of reported intra-oral, extra-oral and general health complaints were slightly lower in the treatment group, but the differences between the groups were not statistically significant. The proportion of individuals currently on sick leave or receiving disability pension was considerably higher in the group of individuals who were excluded from the treatment group compared to the treatment group and the reference group. Participants’ assessments of risks associated with dental amalgam were similar across groups (Table 2).

**Table 2:** Descriptive background data. Background data for the treatment group, the reference group and for patients excluded from the treatment group. Data obtained at the initial examination and from Questionnaire 1 (Q1)

	Treatment group (*n* = 20)	Reference group (*n* = 20)	Excluded from treatment group (*n* = 10)	Data from
Women, *n* (%)	14 (70)	16 (80)	8 (80)	
Age (years) in September 2000, mean (s.d.)	46·9 (6·7)	44·7 (6·5)	52·6 (7·0)	
Education (years), mean (s.d.)	11·5 (3·6)	11·3 (2·8)	10·3 (2·6)	Initial ex.
Reported smoking at initial examination, *n* (%)	4 (20)	7 (35)	3 (30)	Initial ex.
On sick leave or disability pension, *n* (%)	9 (45)	7 (35)	9 (90)	Q1
Regular dental care, *n*/valid *n*^†^ (%)	17/18 (94)	20/20 (100)	6/7 (86)	Q1
Used medication last 12 months, *n* (%)				
Analgesics	13 (65)	13 (65)	7 (70)	Q1
Antidepressants	6 (30)	3 (15)	2 (20)	
Vitamins/dietary supplements	13 (65)	13 (65)	8 (80)	
Participants' assessments of risks associated with dental amalgam, *n* (%)				
Very high	17 (85)	15 (75)	10 (100)	Q1
Medium	3 (15)	4 (20)	–	
Low	–	–	–	
Very low	–	–	–	
Missing	–	1 (5)	–	
Number of amalgam surfaces, mean (s.d.)	36·8 (11·1)	38·0 (11·3)	27·2 (16·3)	Initial ex.
Concentration of mercury, mean (s.d.)				
Blood (nmol L^−1^)	23·5 (10·4)	27·5 (12·5)	33·0 (22·1)	Initial ex.
Urine (nmol L^−1^)	24·0 (17·6)	22·0 (16·4)	21·0 (19·7)	
Urine (nmol per mmol creatinine)	2·7 (1·9)	2·6 (2·7)	2·4 (2·3)	
Self-reported health complaints, mean (s.d.)				
Intra-oral index	8·4 (6·6)	13·0 (12·0)	11·2 (7·2)	Q1
Extra-oral index	6·9 (8·4)	11·0 (9·3)	9·2 (8·0)	
General index	41·5 (16·0)	47·3 (21·2)	42·3 (15·0)	

† Five patients did not answer the question but had started removal of amalgam restorations.

### Comparisons of changes in health complaints in the treatment group and the reference group

Per-protocol comparisons of changes in health complaints, from Questionnaire 1 to the 3-year follow-up in the treatment group and Questionnaire 3 in the reference group, showed that changes in mean index scores for intra-oral and general health complaints were significantly different in the two groups, whereas changes in extra-oral health complaints were not significantly different (Table 3). After adjusting for gender, age and complaint intensity reported in Questionnaire 1, changes in intra-oral and general health complaints remained significantly different, and changes in extra-oral health complaints remained not significantly different (Table 3). Results from intention-to-treat comparisons were in general similar to the results from per-protocol analyses (Table 3). Results from analyses based on data from the reference group after *post hoc* application of exclusion criteria showed no major differences compared with the analyses using all 13 participants from the initial reference group. Unadjusted per-protocol differences in changes in index scores between the treatment group and the reference group after application of exclusion criteria were 8·3 (95% CI: 1·2 to 15·3, *P* = 0·024), 3·6 (95% CI: −3·7 to 10·7, *P* = 0·320) and 19·9 (95% CI: 8·1 to 31·7, *P* = 0·002) for the intra-oral, extra-oral and general indices, respectively.

**Table 3:** Comparisons of changes in health complaints in the treatment group and the reference group. Per-protocol (PP) and intention-to-treat (ITT) comparisons of changes in health complaints from Questionnaire 1 to the 3-year follow-up in the treatment group and Questionnaire 3 in the reference group. Mean changes in index scores and mean differences in changes in index scores (mean changes in the treatment group minus mean changes in the reference group) are given

		Difference Questionnaire 1- to 3-year follow-up†		Unadjusted differences in changes in index scores‡			Adjusted difference in changes in index scores§		
				
	*n*	Mean¶	95% CI	Mean	95% CI	*P*-value*	Mean	95% CI	*P*-value*
Intra-oral index									
Treatment group (PP)	19	3·7	0·5 to 6·9						
Reference group (PP)	13	−4·2	−11·6 to 3·1						
Treatment–reference (PP)				7·9	1·1 to 14·7	0·024	8·1	1·9 to 14·2	0·012
Treatment group (ITT)	20	3·5	0·4 to 6·6						
Reference group (ITT)	20	−0·6	−6·4 to 5·2						
Treatment–reference (ITT)				4·1	−2·3 to 10·5	0·200	6·9	1·3 to 12·4	0·016
Extra-oral index									
Treatment group (PP)	19	1·5	−2·8 to 5·8						
Reference group (PP)	13	−1·8	−7·9 to 4·3						
Treatment–reference (PP)				3·2	−3·7 to 10·2	0·346	5·5	−0·4 to 11·4	0·066
Treatment group (ITT)	20	2·0	−2·2 to 6·2						
Reference group (ITT)	20	−0·6	−4·8 to 3·6						
Treatment–reference (ITT)				2·6	−3·1 to 8·3	0·365	4·3	−1·6 to 10·3	0·145
General index									
Treatment group (PP)	19	9·7	4·4 to 15·0						
Reference group (PP)	13	−8·7	−21·4 to 4·0						
Treatment–reference (PP)				18·4	6·8 to 30·0	0·003	17·4	5·8 to 29·0	0·005
Treatment group (ITT)	20	10·1	5·0 to 15·2						
Reference group (ITT)	20	−2·3	−13·1 to 8·5						
Treatment–reference (ITT)				12·4	0·9 to 23·9	0·036	14·2	2·4 to 26·0	0·020

* Level of significance: *P* < 0·05.

† For the reference group, data from Questionnaire 3 were used.

‡ Independent-sample *t*-test comparing changes in index scores in the treatment group and the reference group.

§ Analysis of covariance of changes in index scores in the treatment group and the reference group, adjusted for gender, age and health complaints from Questionnaire 1.

¶ Positive values indicate reduced health complaints, and negative values indicate increased health complaints.

### Changes in health complaints in the treatment group

**Fig. 3:**
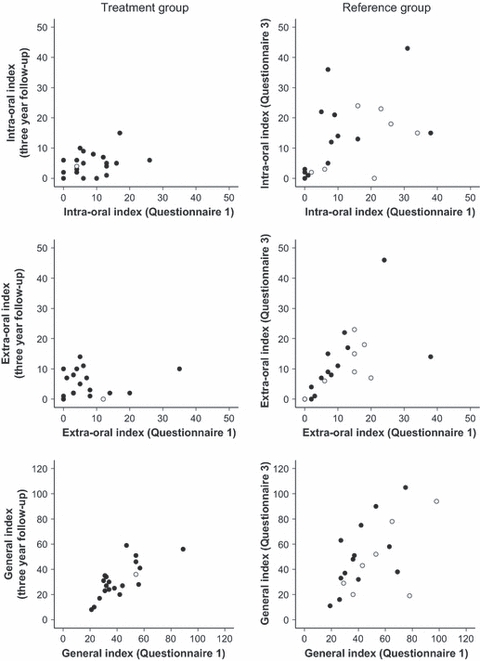
Individual index scores from 3-year follow-up and Questionnaire 3 plotted against scores from Questionnaire 1. Index scores for intra-oral, extra-oral and general health complaints from treatment group (left column) at 3-year follow-up plotted against index scores before amalgam removal (Questionnaire 1). For the reference group (right column), index scores from Questionnaire 3 were plotted against index scores from Questionnaire 1. Data from intention-to-treat analyses (last value carried forward) are marked with grey dots in the diagrams. Results from statistical analyses of data are given in Table 3.

In the treatment group, there were significant reductions in mean index scores for intra-oral and general health complaints from Questionnaire 1 to the 3-year follow-up (Table 3). The reduction in mean index scores for extra-oral health complaints in this period was not significant. Intention-to-treat analysis showed similar results as the per-protocol analysis (Table 3). In the repeated measures analysis (Table 4), data from the pre-treatment examination and all follow-ups were included. Per-protocol repeated measures analysis showed significant overall effects of time for all three index scores. Plots of intra-oral, extra-oral and general index scores from Questionnaire 1 against index scores at 3-year follow-up are given in Fig. 3.

**Table 4:** Repeated measures analysis of changes in health complaints over time. Per-protocol (PP) and intention-to-treat (ITT) repeated measures analysis of changes in health complaints in the treatment group and the reference group. Mean index scores, standard deviations (s.d.) and *P*-values for within-group changes over time are given

		Questionnaire 1	Pre-treatment examination	3-month follow-up	1-year follow-up†	3-year follow-up‡	
							
	*n*	Mean (s.d.)	Mean (s.d.)	Mean (s.d.)	Mean (s.d.)	Mean (s.d.)	*P*-value*
Treatment group							
Intra-oral index (PP)	18	8·6 (6·9)	6·6 (3·8)	6·7 (4·5)	4·7 (5·2)	5·2 (3·8)	0·026
Intra-oral index (ITT)	20	8·4 (6·6)	6·8 (3·7)	6·4 (4·5)	4·8 (5·0)	4·9 (3·8)	0·015
Extra-oral index (PP)	18	6·7 (8·8)	6·7 (7·1)	5·7 (6·4)	2·4 (3·2)	4·8 (4·3)	0·004§
Extra-oral index (ITT)	20	6·9 (8·4)	6·8 (6·8)	5·6 (6·3)	2·8 (3·8)	4·9 (4·4)	0·009§
General index (PP)	18	41·4 (16·4)	42·9 (21·3)	39·0 (24·3)	32·1 (19·2)	31·6 (14·5)	0·001
General index (ITT)	20	41·5 (16·0)	42·7 (20·4)	37·9 (23·2)	31·6 (18·5)	31·4 (13·9)	<0·001
Reference group							
Intra-oral index (PP)	12	11·0 (12·0)	n.a.	n.a.	10·8 (12·8)	15·4 (13·4)	0·245§
Intra-oral index (ITT)	20	13·0 (12·0)	n.a.	n.a.	11·3 (12·4)	13·6 (12·2)	0·246§
Extra-oral index (PP)	12	10·8 (10·6)	n.a.	n.a.	9·4 (11·4)	12·5 (12·6)	0·179§
Extra-oral index (ITT)	20	11·0 (9·3)	n.a.	n.a.	10·0 (10·0)	11·6 (10·8)	0·259§
General index (PP)	12	43·1 (18·1)	n.a.	n.a.	38·3 (23·3)	49·5 (28·5)	0·004§
General index (ITT)	20	47·3 (21·2)	n.a.	n.a.	41·3 (25·2)	49·6 (27·3)	0·004§

n.a., not applicable.

* *P*-value from analysis of variance for repeated measures.

† For the reference group, mean index scores from Questionnaire 2 were used.

‡ For the reference group, mean index scores from Questionnaire 3 were used.

§ Wilks’ Lambda.

### Changes in health complaints in the reference group

In the reference group, there was a slight, but not statistically significant, increase in mean index scores for intra-oral, extra-oral and general health complaints from Questionnaire 1 to Questionnaire 3 (Table 3). Intention-to-treat analysis showed no significant changes in mean index scores from Questionnaire 1 to Questionnaire 3 (Table 3). Data from Questionnaire 2 were included in the repeated measures analysis (Table 4). Per-protocol analysis of changes in mean index scores over time showed a significant overall effect of time for general health complaints. Plots of intra-oral, extra-oral and general index scores from Questionnaire 1 against index scores from Questionnaire 3 are given in Fig. 3.

### Mercury concentration in serum and urine

**Fig. 4:**
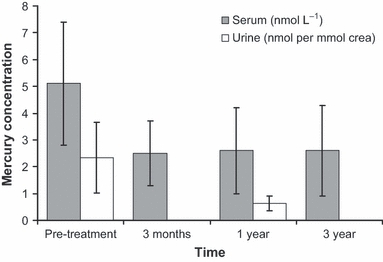
Mean mercury concentration in serum and urine at pre-treatment examination and at follow-up after removal of amalgam fillings. Mean mercury concentration (and s.d.) in serum (nmol L^−1^) and urine (nmol per mmol creatinine) at pre-treatment examination and at follow-up after removal of amalgam fillings. Mercury concentration in both serum and urine was significantly reduced after amalgam removal (*P* < 0·001, and *P* = 0·004, respectively).

There was a significant decrease in mercury concentration in serum and urine following the removal of amalgam fillings. After removal of the fillings, the mean serum concentration was reduced to half the concentration at pre-treatment, and the mean concentration in urine was reduced to about one-fourth of the pre-treatment concentration (Fig. 4).

### Changes in health complaints related to changes in mercury concentration in serum

Secondary explorative analyses of correlations between reduction in mercury in serum and reduction in health complaints 3 years after treatment showed positive but not significant correlations. Pearson correlation coefficients were 0·320, 0·193 and 0·127 for correlations between reduction in mercury in serum and reduction in intra-oral, extra-oral and general indices, respectively. Corresponding *P*-values were 0·182, 0·428 and 0·604 (*n* = 19), leaving no statistically significant support for mercury as a cause of the complaints.

### Adverse events

Seven participants in the treatment group experienced increased health complaints in connection with removal of amalgam fillings. Laboratory tests of blood samples collected within a few days after the treatment session showed values within reference intervals. Health complaints reported in connection with amalgam removal were gastric pain, pain in joints and muscles, oral ulcers, sore throat, pain in legs, hands and feet, dizziness, tachycardia, nausea, diarrhoea, depression, fatigue, chills, burning sensations in the face, cold hands, increased blood pressure and submandibular lymphadenopathy. The increase in complaints was transient and disappeared within a week or two.

## Discussion

The aim of this study was to investigate long-term changes in subjective health complaints after the removal of all amalgam fillings in a group of patients who attributed health complaints to amalgam fillings. The main finding was that the long-lasting reductions in intra-oral and general health complaints in the treatment group were significantly different from the change in the reference group, in which there were no long-lasting reductions.

In the treatment group, intra-oral and general health complaints were significantly reduced 3 years after completed replacement of amalgam fillings. Reductions in subjective health complaints after replacement of amalgam fillings have also been found in previous studies (24, 25). The reference group received no intervention, and no improvement in health complaints was found. This is in agreement with data from patients with health complaints attributed to dental restorations, mainly dental amalgam, who did not change the restorations to other materials (8).

It is necessary to consider several factors that may have influenced the results. First, there has been a reduced exposure to mercury in the treatment group. Previous studies have established that people with amalgam fillings have higher concentrations of mercury in blood, plasma, urine and body organs than people without amalgam fillings (12, 15, 17–19, 31). The finding of reduced levels of mercury in serum and urine in the present study is in agreement with data from several studies showing that replacement of amalgam fillings leads to reduced levels of mercury in blood, plasma and urine (14, 32, 33). Despite this, studies investigating the relationship between amalgam fillings and reported health complaints have not found positive correlations between number of amalgam fillings and number of reported complaints (9, 18), indicating that if there is a causal relationship between amalgam fillings and health effects, there is not a simple dose–response relationship between exposure to amalgam fillings and reported health complaints. In a recently published study on health effects after removal of amalgam fillings (34), correlations between amalgam-filled surfaces and symptom scores were not statistically significant. However, positive moderate correlations were found between mercury levels in both plasma and urine and subjective health complaints, and between reductions in mercury levels in these media and reductions in subjective health complaints (34). In the present study, we found positive but not significant correlations between reduction in mercury concentration in serum and reductions in subjective health complaints, which may be in agreement with the analyses presented in (34). It is possible that some individuals are highly sensitive to mercury from dental amalgam and may benefit from reduced exposure (35).

The reference group received no treatment and was only followed up by questionnaires sent by post. This makes it difficult to untangle the effects of the general care associated with amalgam replacement and follow-ups in the treatment group from the effects of the amalgam replacement itself. Follow-ups in the treatment group were carried out by health personnel with both time and motivation to listen to and understand the patients’ experiences. This may have contributed to the reduction in reported subjective health complaints as patient-centred communication has been shown to be associated with improved patient health outcomes (36, 37). In addition, participants in the treatment group no longer had to worry about possible adverse effects from their amalgam fillings. This may also have played a part in the reduction in health complaints as worry has been found to lead to increased monitoring of complaints, which again may lead to an increased feeling of ill health (38). Even so, replacement of amalgam fillings will usually take place in a treatment context where factors like these are present and, thus, potentially might influence the treatment results. Participants’ belief in amalgam replacement as an effective treatment (39) and gratitude in relation to having the replacement covered by project funds could possibly have resulted in a response bias towards reporting reduced health complaints. However, it is not likely that the participants would remember how they responded to the scales in the questionnaires several years ago. Factors mentioned above are linked to components related to placebo (expectations, conditioning, learning, memory, motivation, somatic focus, reward, anxiety reduction and meaning), as defined as a genuine psychobiological event attributable to the overall therapeutic context (40). In this context, it is also possible that for some patients, the presence of amalgam fillings has been associated with a nocebo effect. Removal of amalgam fillings could therefore result in a discontinuation of this effect and consequently lead to a reduction in reported health complaints.

Reduction in intra-oral health complaints may have been influenced by general effects of the dental treatment received during the amalgam replacement process. It does, however, seem unlikely that an effect of a generally improved dental health should be prominent 3 years after completed replacement, given that patients with need for complicated dental rehabilitation were excluded from the treatment group and that the removed amalgam fillings were described as sound and well-functioning.

Participants included in this study were recruited from patients referred to the Dental Biomaterials Adverse Reaction Unit. Consequently, participants are not representative of all patients with health complaints attributed to amalgam fillings. Not all patients with health complaints attributed to dental amalgam are referred to this unit. Some patients are directly treated by their own dentist or general practitioner or seek help from practitioners of alternative medicine. Despite lack of objective signs of adverse reactions to dental amalgam, some patients nevertheless have all their amalgam fillings removed of their own accord because they are concerned about possible adverse effects of mercury released from amalgam fillings. The participants included in this study had not removed all amalgam fillings, either because they accepted that there were no indications for amalgam removal or because they did not have the financial means necessary for amalgam removal. Thus, the treatment group is not directly comparable with patients who remove amalgam restorations of their own accord (23).

The study was designed as a before-and-after study with a comparison group (reference group). Comparisons between the reference group and the treatment group must be interpreted with caution. Even though power calculations showed acceptable power of the study, the sample size is small and the results should be considered in context with results from comparable studies (8, 24, 25, 41). A larger sample size could provide more precise estimates and less-wide confidence intervals. In addition, there may be unknown factors that influence reporting of health complaints over time in the groups. Another limitation could be that as the outcome is based on the participants’ reporting of health complaints, the study is open for response bias in both the treatment group and the reference group.

In the treatment group, all 20 participants completed replacement of amalgam fillings, and 19 of the participants were able to attend the 3-year follow-up. In the reference group, seven of the 20 participants were lost to follow-up or excluded because of completed removal of amalgam fillings (Fig. 1). The response rate in the reference group was influenced by the fact that only two reminders, by letter, is allowed by the Regional Committee for Medical Research Ethics. This is in line with the standards used by the Norwegian National Committee for Medical and Health Research Ethics. As there were no major differences between the results from the per-protocol analyses and the intention-to-treat analyses, we assume the potential bias from non-random dropout of participants or exclusion of ‘protocol violators’ (participants in the reference group who removed amalgam during the study) had no major impact on the result.

Exclusion criteria were initially not applied in the reference group. The clinical examination necessary to fully apply these criteria could potentially lead to a renewed focus on amalgam fillings as a possible cause of ill health, thus increasing the risk of participants in the reference group initiating amalgam removal of their own accord. As no intervention was planned for the reference group, the participants were not asked to undergo a clinical examination. The patients excluded from the treatment group were, based on their responses to Questionnaire 1, quite similar to the treatment group and the reference group, with the exception of per cent on sick leave or disability pension. For this variable, exclusion of the 10 patients resulted in a more equal occupational status for the treatment group and the reference group (Table 2). Changes in health complaints in the treatment group were compared with changes in both the initial reference group and changes in the reference group after *post hoc* application of exclusion criteria. No major differences were found between the two comparisons. However, as there was no clinical pre-treatment examination of patients in the reference group, there could still be differences between the groups. The bias from differences between the groups at study start is expected to be limited.

Treatment of patients with subjective health complaints attributed to amalgam fillings should only be considered after a thorough medical and dental examination has been carried out and other causes for the complaints have been eliminated or adequately treated (42). The results from the present study, and other studies investigating the effects of amalgam replacement, indicate that replacement of amalgam fillings is associated with reductions in subjective health complaints at group level. The mechanisms behind this are not known, and other treatment options than amalgam replacement should also be considered. In a recent randomised clinical trial, all investigated treatments (amalgam removal, amalgam removal plus biological detoxification and health promotion without amalgam removal) resulted in clinically relevant reductions in health complaints (25). When considering replacement of intact amalgam fillings, potential benefits must be balanced with risks associated with the dental treatment (e.g. tooth fractures or endodontic complications). When removing amalgam fillings, measures should be taken in order to minimise exposure to mercury for both patients and dental personnel (29, 42).

The results from the present study indicate that the replacement of amalgam fillings was associated with reductions in subjective health complaints at group level. The mechanisms behind this remain to be identified. Reduced exposure to mercury, patient-centred treatment and follow-ups, and elimination of worry are factors that may have influenced the results. In this study, we investigated changes in index scores. More knowledge is needed about changes in specific complaints included in the index scores after replacement of amalgam fillings, and a characterisation of the treatment group in this respect is warranted.

## Footnotes

* SPSS Inc., Chicago, IL, USA.
